# The Prevalence and Significance of Incidental Positron Emission Tomography Findings in the Brain Using Radiotracers Other than [^18^F]FDG: A Systematic Review and Meta-Analysis

**DOI:** 10.3390/diagnostics15101204

**Published:** 2025-05-09

**Authors:** Cesare Michele Iacovitti, Barbara Muoio, Domenico Albano, Alessio Rizzo, Marco Cuzzocrea, Gaetano Paone, Giorgio Treglia

**Affiliations:** 1 Division of Nuclear Medicine, Imaging Institute of Southern Switzerland, Ente Ospedaliero Cantonale, 6500 Bellinzona, Switzerland; cesaremichele.iacovitti@eoc.ch (C.M.I.); marco.cuzzocrea@eoc.ch (M.C.); gaetano.paone@eoc.ch (G.P.); giorgio.treglia@eoc.ch (G.T.); 2 Division of Medical Oncology, Oncology Institute of Southern Switzerland, Ente Ospedaliero Cantonale, 6500 Bellinzona, Switzerland; 3 Faculty of Biomedical Sciences, Università della Svizzera italiana, 6900 Lugano, Switzerland; 4 Department of Nuclear Medicine, ASST Spedali Civili di Brescia and University of Brescia, 25121 Brescia, Italy; domenico.albano@unibs.it; 5 Department of Nuclear Medicine, Candiolo Cancer Institute, FPO-IRCCS, 10060 Turin, Italy; alessio.rizzo@ircc.it; 6 Faculty of Biology and Medicine, University of Lausanne, 1015 Lausanne, Switzerland

**Keywords:** PET, positron emission tomography, incidental, incidentalomas, brain tumors, brain lesions, nuclear medicine, meningioma, brain metastases, neuroimaging, neuro-oncology

## Abstract

**Background**: Incidental brain imaging findings could be clinically relevant, and advancements in molecular imaging could lead to their more frequent identification. The aim of this review is to establish the prevalence and clinical significance of brain incidentalomas at PET (BIPs) using radiotracers other than [^18^F]FDG. **Methods**: A comprehensive literature search of studies about BIPs was carried out. Four different databases (PubMed/MEDLINE, EMBASE, the Cochrane library, and Google Scholar) were screened up to December 2024. Only original articles about BIPs using radiotracers other than [^18^F]FDG were selected. A proportion meta-analysis of the prevalence of BIPs was carried out using a random-effects model. **Results**: Fourteen studies were included in the review, using somatostatin receptor (SSTR) PET (*n* = 6), radiolabeled choline PET (*n* = 5), prostate-specific membrane antigen (PSMA) ligands PET (*n* = 1), [^18^F]Fluciclovine PET (*n* = 1), and [^18^F]FDOPA PET (*n* = 1). The pooled prevalence of BIPs was 4.6% for SSTR PET, 1.1% for choline PET, 1.2% for PSMA ligands PET, 2.5% for [^18^F]Fluciclovine PET, and 3.9% for [^18^F]FDOPA PET. When BIPs were further evaluated using MRI, meningiomas were the most frequent lesions detected, but both benign and malignant lesions could be incidentally diagnosed. **Conclusions**: BIPs using radiotracers other than [^18^F]FDG are not rare, in particular at SSTR PET, further justifying the extension of PET scans to the brain when radiotracers other than [^18^F]FDG are used. When detected, a BIP should be further evaluated using brain MRI. Both benign and malignant lesions could be incidentally detected in the brain. Further studies are warranted to better clarify the clinical impact of BIP detection.

## 1. Introduction

Incidental imaging findings or incidentalomas are unexpected lesions diagnosed in patients undergoing imaging for an unrelated reason. The increasing use of imaging methods coupled with better image resolution is driving a surge in incidentalomas in different organs [1,2].

Incidental imaging findings could be clinically relevant, and advancements in molecular imaging could lead to their being more frequently identified than in morphological imaging, as functional abnormalities may precede morphological changes [1,2]. In this regard, positron emission tomography (PET) using different radiopharmaceuticals is an increasingly used molecular imaging technique for oncological and non-oncological indications [3]. PET can be coupled with computed tomography (PET/CT) or magnetic resonance imaging (PET/MRI) as hybrid imaging, and different radiotracers evaluating the expression of several metabolic patterns or receptors could be used [3].

The prevalence and nature of incidentalomas may vary depending on the anatomical site and the imaging modality used. In respect of morphological brain imaging, recent analyses, including MRI, have already clarified the prevalence and clinical significance of brain incidental findings [4,5,6]. The prevalence of incidental findings with brain MRI is around 4%, and it is even higher when white matter hyperintensities are included. Some of these brain incidental findings are clinically significant, necessitating further investigation or treatment and resulting in increased costs to healthcare systems as well as increased patient anxiety [4,5,6].

Conversely, the prevalence of brain incidental findings at functional imaging including PET with different tracers has not been fully evaluated. To date, PET with fluorine-18 fluorodeoxyglucose ([^18^F]FDG), a radiolabeled glucose analogue, is the most used PET imaging method. However, this imaging method has proved to be problematic in the evaluation of brain lesions due to a low lesion-to-background uptake ratio, as the brain demonstrates increased glucose consumption and consequently high physiologic tracer uptake, which reduces the sensitivity of the method for brain lesion detection and delineation [7]. Only a meta-analysis has assessed the pooled prevalence and clinical significance of incidental [^18^F]FDG uptake in the pituitary, demonstrating that this prevalence is 0.33% and adenomas are the most frequent lesions [8].

**Figure 1:**
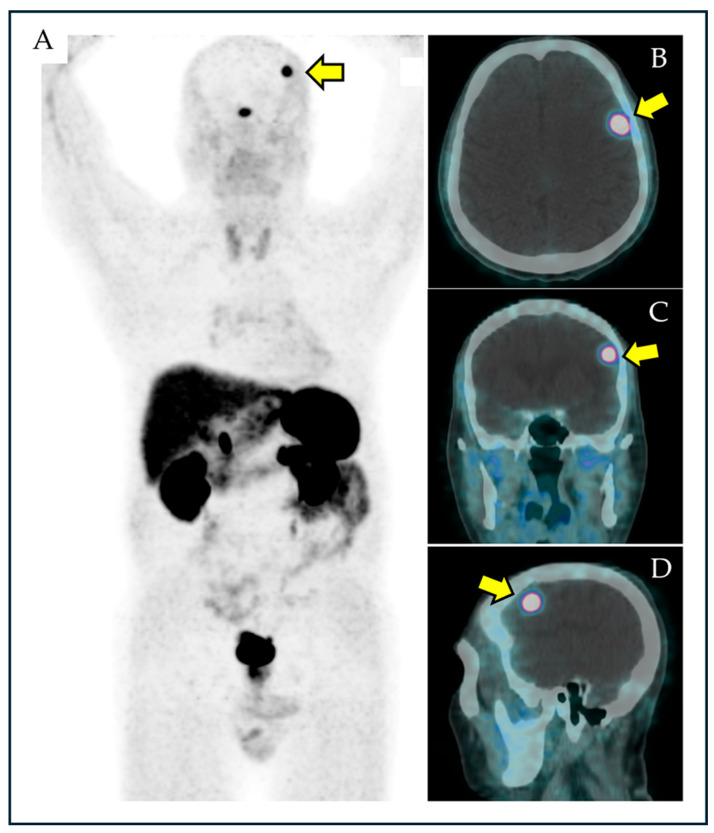
A case of brain incidental finding (yellow arrow) at somatostatin receptor PET/CT with [^68^Ga]Ga-DOTA-peptides detected at maximum-intensity projection PET image (**A**), axial (**B**), coronal (**C**), and sagittal (**D**) PET/CT images.

PET/CT and PET/MRI can be currently performed with other tracers beyond [^18^F]FDG. These PET tracers are usually characterized by an absence of low physiological tracer uptake in the brain, thus allowing better detection of incidental brain lesions [7]. An example of a brain incidentaloma with PET using other tracers beyond [^18^F]FDG is reported in Figure 1.

The aim of this review is therefore to evaluate the current literature to establish the prevalence and the clinical significance of brain incidentalomas at PET (BIPs) using radiotracers other than [^18^F]FDG. Our hypothesis is that the prevalence and clinical significance of BIPs could be quite different based on the PET tracer used.

## 2. Materials and Methods

### 2.1. Review Protocol, Working Group, and Review Question

This review article was written following a predefined protocol [9], and it was reported according to the PRISMA statement [10]. The protocol was not published on PROSPERO or other databases, as this is suggested but not mandatory according to the PRISMA statement [10].

The working group was composed of one neuro-oncologist (B.M.), five senior nuclear medicine physicians (D.A., A.R., M.C., G.P., and G.T.), and one junior nuclear medicine physician (C.M.I.). Four co-authors have extensive experience in systematic reviews and meta-analyses on PET (B.M., D.A., A.R., and G.T.).

The first step of this review was to formulate a clear review question clearly defining patients, interventions, and outcomes. The review question was the following: “Which is the prevalence and clinical significance of brain incidentalomas at PET using other radiotracers beyond [^18^F]FDG?”.

### 2.2. Search Strategy

A comprehensive literature search of studies about BIPs was carried out by three review authors independently (C.M.I., B.M., and G.T.). Four different databases (PubMed/MEDLINE, EMBASE, the Cochrane library, and Google Scholar) were screened up to 31 December 2024. The following search string combining several free text words related to the review question was used: (A) “brain” OR “cerebral” OR “CNS” OR “central nervous system” AND (B) “incidental” OR “incidentaloma*” OR “incidental*” OR “unexpected” OR “unusual” AND (C) “PET” OR “positron”.

No beginning date limit or language restrictions were used during the literature search. The references of the retrieved articles were also screened as a means of searching for additional studies.

### 2.3. Study Selection

Three review authors independently performed the study selection (C.M.I., B.M., and G.T.) applying the predefined inclusion and exclusion criteria according to the review question. Inclusion criteria: studies or subsets of studies investigating the prevalence and significance of BIPs using other radiotracers beyond [^18^F]FDG. Exclusion criteria: (a) articles outside the scope of this review or not containing information on the prevalence of BIP; (b) review articles, editorials, comments, letters, and conference proceedings in the field of interest of this review; (c) case reports in the field of interest of this review.

Titles and abstracts of the records retrieved using the predefined search string in the selected databases were screened. After the exclusion of non-eligible records, the full texts of the potential eligible articles were downloaded and screened. Finally, studies were included in the review after a virtual consensus meeting. Articles included in the systematic review were included in the meta-analysis only when there was sufficient data to calculate the prevalence of BIPs using radiotracers other than [^18^F]FDG (e.g., when both the number of patients undergoing PET and the number of BIPs were specified in the included articles).

### 2.4. Data Extraction and Quality Assessment

Three review authors (C.M.I., B.M., and G.T.) independently extracted the following information from the selected articles using predefined data collection forms: basic study characteristics, patient characteristics, technical aspects, and outcome data.

The overall quality of the studies included in this systematic review was evaluated online using the NIH quality assessment tools [11].

### 2.5. Statistical Analysis

The pooled prevalence of BIPs using radiotracers other than [^18^F]FDG was calculated for each radiopharmaceutical, when sufficient studies were available, through a patient-based proportion meta-analysis using a random-effects model that considered the variability between studies. Studies were excluded from the meta-analysis in cases of possible patient data overlap among studies; in such cases, only the most complete study was included in the meta-analysis. Pooled data were presented with 95% confidence interval (95%CI) values. Heterogeneity was estimated through the inconsistency index (I^2^) [9]. OpenMeta[Analyst] (version 1.0 for Windows) was used as free open-source statistical software.

## 3. Results

### 3.1. Literature Search

**Figure 2:**
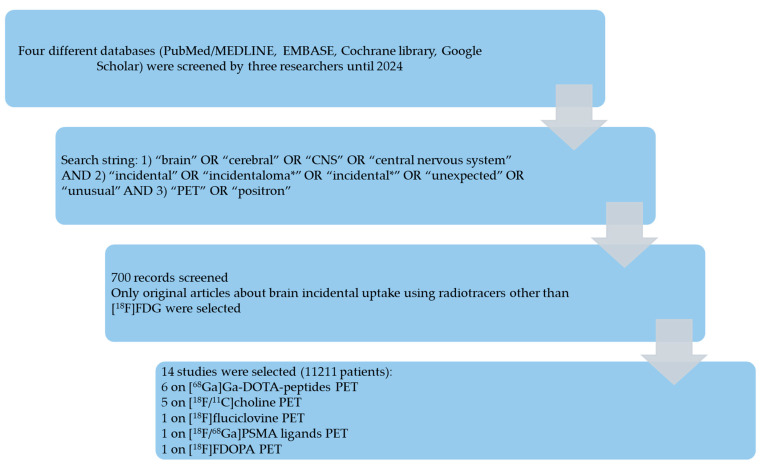
Results of the literature search.

Figure 2 summarizes the results of the literature search. A total of 700 records were identified using the selected databases. Titles and abstracts of these records were screened; 14 studies were finally included in the systematic review [12,13,14,15,16,17,18,19,20,21,22,23,24,25], and no additional studies were found by screening the reference list of the retrieved articles.

Six studies evaluated the prevalence of BIPs with [^68^Ga]Ga-DOTA-peptides [13,14,15,18,21,23], five studies evaluated the prevalence of BIPs using radiolabeled choline [17,19,22,24,25], and only one study was available for radiolabeled prostate-specific membrane antigen (PSMA) ligands [12], [^18^F]Fluciclovine [16], and [^18^F]FDOPA [20], respectively. A meta-analysis was feasible for studies about [^68^Ga]Ga-DOTA-peptides and radiolabeled choline.

### 3.2. Qualitative Synthesis

Table 1 summarizes the main findings about original studies on BIPs with the use of radiotracers other than [^18^F]FDG on 11,211 patients. The quality of all included studies was moderate according to the NIH quality assessment tool.

#### 3.2.1. [^68^Ga]Ga-DOTA-Peptides (Somatostatin Receptor PET)

Six studies evaluated BIPs at somatostatin receptor PET (using [^68^Ga]Ga-DOTA-peptides), usually performed for the evaluation of neuroendocrine neoplasms [13,14,15,18,21,23]. The prevalence of BIPs ranged from 1.8% to 11.2% of all PET scans (PET/CT or PET/MRI). Notably, when further evaluated with brain MRI, most of the BIPs at somatostatin receptor PET were found to be meningiomas [13,14,15,18,21,23]. Less frequently, BIPs at somatostatin receptor PET corresponded to pituitary adenomas, hemangiomas, venous anomalies and structures, benign fibro-osseous lesions, skull base paragangliomas, schwannomas, brain metastases of neuroendocrine tumors, and, rarely, brain metastases from non-neuroendocrine tumors [13,14,15,18,21,23]. Cases with a concordant lesion-type prediction of meningioma between MRI and somatostatin receptor PET displayed a significantly higher tracer uptake value at somatostatin receptor PET compared with cases with a discordant prediction of meningioma [13].

#### 3.2.2. Radiolabeled Choline ([^18^F/^11^C]Choline)

Five studies evaluated BIPs at radiolabeled choline PET (using [^18^F/^11^C]choline), usually performed for the evaluation of prostate cancer [17,19,22,24,25]. The prevalence of BIPs ranged from 0.5% to 2.5% of all PET scans (PET/CT or PET/MRI). Notably, when further evaluated with brain MRI, most of the BIPs at radiolabeled choline PET were found to be meningiomas [17,19,22,24,25]. Less frequently, BIPs at radiolabeled choline PET corresponded to gliomas or other brain tumors, including brain metastases [19,24].

#### 3.2.3. Radiolabeled PSMA Ligands ([^18^F/^68^Ga]PSMA Ligands)

One recent study evaluated BIPs at radiolabeled PSMA ligands PET (using [^18^F/^68^Ga]PSMA ligands), usually performed for the evaluation of prostate cancer [12]. The prevalence of BIPs was 1.2% of all PET scans (PET/CT or PET/MRI). Notably, when further evaluated with brain MRI, about half of the BIPs at radiolabeled PSMA ligands PET were found to be meningiomas [12]. However, BIPs at radiolabeled PSMA ligands PET may also correspond to brain metastases in about 40% of cases and less frequently to pituitary adenomas and epidermal inclusion cysts [12]. From the results that emerged from the study, it seems that the tracer uptake values cannot be used to differentiate among the different etiologies of BIPs [12].

#### 3.2.4. [^18^F]Fluciclovine

One study evaluated BIPs at [^18^F]Fluciclovine PET, usually performed for the evaluation of prostate cancer in biochemical recurrence [16]. The prevalence of BIPs was 2.5% of all PET scans (PET/CT or PET/MRI). Notably, when further evaluated with brain MRI, most of the BIPs at [^18^F]Fluciclovine PET were found to be meningiomas. Less frequently, BIPs at [^18^F]Fluciclovine PET corresponded to other benign lesions or brain metastases [16].

#### 3.2.5. [^18^F]FDOPA

One study evaluated BIPs at [^18^F]FDOPA PET, usually performed for the evaluation of neuroendocrine tumors or for the diagnostic management of movement disorders or brain lesions [20]. The prevalence of BIPs, excluding patients undergoing PET for brain lesion or brain tumor evaluation, was 3.9%. When further evaluated with brain MRI, BIPs at [^18^F]FDOPA PET were found to be benign tumor lesions such as pituitary adenomas or ependymomas [20].

### 3.3. Quantitative Synthesis

Meta-analysis on the prevalence of BIPs was performed only for somatostatin receptor PET (six studies) and radiolabeled choline PET (three studies excluding those with possible patient data overlap). The pooled prevalence of BIPs was 4.6% (95%CI: 2.6–6.6%) and 1% (95%CI: 0.5–1.5%), respectively. Moderate statistical heterogeneity was found in the meta-analysis on somatostatin receptor PET (I^2^ > 50%), but not in the meta-analysis on radiolabeled choline PET (I^2^ < 50%).

## 4. Discussion

### 4.1. Literature Data

To the best of our knowledge, this is the first comprehensive review and pooled analysis summarizing the prevalence and significance of BIPs using PET tracers other than [^18^F]FDG.

Notably, we have included only original articles in our comprehensive review excluding case reports, since case reports are affected by several biases reducing the quality of evidence (i.e., reporting bias and patient selection bias). This is in line with guidelines that suggest removing case reports from consideration in systematic reviews, in part due to the challenges in evaluating the internal validity of these study designs [26].

Compared to [^18^F]FDG, other PET tracers are characterized by absent or low physiological tracer uptake in the brain, thus allowing an easier detection of BIPs due to low uptake in the background [7]. We have found that most of the literature data on BIPs are focused on [^68^Ga]Ga-DOTA-peptides and radiolabeled choline, and less information is currently available for other PET tracers.

Somatostatin receptor PET is currently used for the diagnostic management of several neuroendocrine tumors [27]. The brain is a quite frequent site of incidental findings at somatostatin receptor PET using [^68^Ga]Ga-DOTA-peptides, whereas the pituitary gland is a site of physiological tracer uptake [28]. In our analysis, we found that the pooled prevalence of BIPs at somatostatin receptor PET was 4.6%, and it was significantly higher compared to other PET tracers assessed. The most frequent etiologies of BIPs at somatostatin receptor PET, when further evaluated by MRI, were meningiomas [13,14,15,18,21,23]. These findings are not surprising, as meningiomas are the most frequent subtype of brain tumor, and they are usually characterized by the increased expression of somatostatin receptors, thus explaining the high uptake of radiolabeled somatostatin analogues such as [^68^Ga]Ga-DOTA-peptides [29]. Recent international guidelines also recommend using somatostatin receptor PET to evaluate meningiomas in specific settings [30]. However, albeit less frequently, other brain lesions beyond meningiomas, including benign and malignant lesions overexpressing somatostatin receptors, may be the cause of BIPs at somatostatin receptor PET [13,14,15,18,21,23], and brain MRI can help to clarify these findings. Interestingly, the overexpression of somatostatin receptors by some BIPs also paves the way for a therapeutic option using radioligand therapy with radiolabeled somatostatin analogues (the theragnostic approach) [18].

Radiolabeled choline PET has some indications in oncology for evaluating prostate cancer and other tumors [31,32]. Interestingly, BIPs are significantly less frequent at radiolabeled choline PET compared to somatostatin receptor PET, as demonstrated by our pooled analysis (pooled prevalence of 1.1% vs. 4.6%, respectively, without an overlap in the 95%CI values). Notably, meningiomas are the most frequent cause of BIPs at radiolabeled choline PET [17,19,22,24,25]; however, compared to [^68^Ga]Ga-DOTA-peptides, this finding is not sufficient to justify the use of radiolabeled choline PET in the management of meningiomas. Furthermore, other primary brain tumors or brain metastases may demonstrate a high radiolabeled choline uptake [19,24]. The reason for the increased radiolabeled choline uptake in these tumors is poorly documented and may involve an increase in cell membrane synthesis and turnover [17,33].

PET/CT or PET/MRI with radiolabeled PSMA ligands is currently increasingly used for oncological indications, in particular for the management of prostate cancer [34]. However, several tumors beyond prostate cancer may overexpress PSMA in their microenvironment (e.g., neovasculature), causing an increased uptake of radiolabeled PSMA ligands [35,36]. Furthermore, incidental findings at radiolabeled PSMA ligands PET have already been extensively reported [37,38,39,40,41,42]. In this context, it is surprising that only one original article has assessed the prevalence of BIPs at radiolabeled PSMA ligands PET, reporting a prevalence of 1.2% [12]. Meningiomas are reported as the etiology of BIPs at radiolabeled PSMA ligands PET in about 50% of cases. However, the percentage of malignant brain lesions as the etiology of BIPs increases with radiolabeled PSMA ligands compared to other PET tracers. Notably, BIPs at radiolabeled PSMA ligands PET may represent previously unknown prostate cancer metastases in a significant percentage of cases (about 40%) even in the absence of systemic disease [12,43]. The early detection of occult brain metastases may obviously change the management of prostate cancer patients in some cases [43]. Interestingly, the increased expression of PSMA ligands in brain tumors also opens the way to a therapeutic option with radioligand therapy using radiolabeled PSMA ligands [44].

PET/CT or PET/MRI with [^18^F]Fluciclovine is currently used for the management of prostate cancer [45], and potential pitfalls and incidental findings have been previously evaluated [46]. However, only one study assessed the prevalence of BIPs at [^18^F]Fluciclovine PET, reporting a prevalence of 2.5% [16]. Notably, meningioma is the most frequent etiology of BIPs at [^18^F]Fluciclovine PET [16]; however, compared to [^68^Ga]Ga-DOTA-peptides PET, this finding is not sufficient to justify the use of [^18^F]Fluciclovine PET in the management of meningiomas. Furthermore, other primary brain tumors or brain metastases may demonstrate high [^18^F]Fluciclovine uptake [16]. The reason for the increased [^18^F]Fluciclovine uptake in these tumors is poorly documented and may involve an increase in amino acid metabolism.

Lastly, only one study evaluated BIPs at [^18^F]FDOPA PET [20], usually performed for the evaluation of neuroendocrine tumors or for the diagnostic management of movement disorders or brain lesions [47]. The prevalence of BIPs, excluding patients undergoing PET for brain lesion or brain tumor evaluation, was 3.9%, and all the BIPs were found to be benign tumors. However, the low number of BIPs at [^18^F]FDOPA PET prevents us from providing a reliable conclusion on the clinical significance of these incidental findings. The same is valid for other PET tracers beyond those described above, because only case reports are available in the literature on BIPs with these tracers, and they are excluded from our review for the reasons already mentioned.

Notably, beyond pituitary adenomas [8], certain hypermetabolic incidental brain lesions (such as lymphomas, high-grade gliomas, and some metastases) are well detectable with [^18^F]FDG PET [48,49,50,51,52]. Incidental brain findings based on hypometabolism at [^18^F]FDG PET could also be of clinical relevance [49,50,51,52,53,54]. Unfortunately, extension of [^18^F]FDG PET to the brain is performed only in selected cases in clinical practice due to the physiological radiopharmaceutical uptake in the brain; therefore, the real prevalence of BIP findings at [^18^F]FDG PET cannot be estimated with precision.

Overall, we can state that BIPs with PET tracers beyond [^18^F]FDG are not so rare, taking into account the available literature data, with a pooled prevalence ranging from around 1% to 5%, thus justifying an extension of the PET scan to the brain when other PET tracers than [^18^F]FDG are used. Furthermore, brain MRI should be always suggested in the PET report to further evaluate BIPs when they are detected, because both benign and malignant lesions are found among BIPs. Meningioma is the most frequent etiology of BIPs when PET tracers beyond [^18^F]FDG are used.

### 4.2. Limitations and Suggestions for Future Research

Several limitations of our analysis should be acknowledged. First, a significant heterogeneity and variability among the included studies is present, most likely due to differences in patient characteristics and imaging protocols and indications among the included studies. Publication bias may also be present, even if we have excluded case reports from the analysis. We have not assessed the publication bias through funnel plots due to the low number of studies included in the analyses for each PET tracer. Another limitation of our analysis is that we have not taken into account the different blood–brain barrier (BBB) permeability of the different PET radiotracers [55]; different BBB permeability can theoretically result in different a clinical impact of BIPs with each PET radiotracer. Lastly, for some PET tracers, we have limited data, which hampers our ability to obtain clear information on the prevalence and clinical significance of BIPs.

As a first suggestion for future research, we recommend that further studies should be conducted on the prevalence and clinical significance of BIPs with other tracers than [^68^Ga]Ga-DOTA-peptides and radiolabeled choline. Second, studies evaluating possible changes in the management of BIP detection with PET imaging are strongly recommended. Third, long-term clinical outcomes of patients with BIPs could be assessed in future studies. Lastly, it would be valuable to obtain specific analyses taking into account the advantage for the brain of BIP detection with a PET scan extension over the additional radiation exposure to the brain from extended CT acquisition, technical constraints related to increased scanner time, and the need for specific neuroimaging expertise among nuclear medicine physicians.

## 5. Conclusions

BIPs using radiotracers other than [^18^F]FDG are not rare, in particular at somatostatin receptor PET. This further justifies the extension of PET scans to the brain when radiotracers other than [^18^F]FDG are used. When detected, a BIP should be further evaluated using brain MRI. Both benign and malignant lesions could be incidentally detected in the brain, even if the most frequent lesions are meningiomas. Further studies are warranted to better clarify the clinical impact of BIP detection.

## Figures and Tables

**Table 1:** Main findings of studies on incidental brain findings at PET (BIPs) with different radiopharmaceuticals.

PET Tracer	Authors	Year	Country	Patients with PET Scans	Patients with BIPs	Patients with Suspicious Meningioma Among Those with BIPs	Prevalence of BIPs	Pooled Prevalence of BIPs
[^68^Ga]Ga-DOTA-peptides	Ensign et al. [13]	2023	USA	3154	145	123 (85%)	4.6%	4.6%
	Albano et al. [15]	2022	Italy/Switzerland	430	48	38 (79%)	11.2%	
	Umana et al. [14]	2022	Italy	1000	18	10 (56%)	1.8%	
	Parghane et al. [18]	2019	India	500	12	6 (50%)	2.4%	
	Cleary et al. [21]	2016	UK/Australia	313	22	21 (95%)	7.0%	
	Kuyumcu et al. [23]	2013	Turkey	120	3	1 (33%)	2.5%	
[^68^Ga/^18^F]PSMA ligands	McLaughlin et al. [12]	2023	USA	2763	33	16 (48%)	1.2%	1.2%
[^18^F/^11^C]Choline	Roland et al. [17]	2020	France	368	2	2 (100%)	0.5%	1%
	Calabria et al. [19]	2017	Italy	1000	14	12 (86%)	1.4%	
	Calabria et al. [22] *	2014	Italy	300	6	6 (100%)	2.0%	
	Schillaci et al. [24] *	2010	Italy	80	2	1 (50%)	2.5%	
	Fallanca et al. [25]	2009	Italy	402	4	4 (100%)	1%	
[^18^F]Fluciclovine	Baiomy et al. [16]	2021	USA	566	14	10 (71%)	2.5%	2.5%
[^18^F]FDOPA	Calabria et al. [20]	2016	Italy	215 (77 **)	3	0 (0%)	3.9%	3.9%

Legend: * included in the systematic review but not in the meta-analysis for possible patient data overlap with the study of Calabria et al. [19]; ** when patients undergoing PET for brain lesion or brain tumors evaluation were excluded; BIPs = brain incidentalomas at PET.

## Data Availability

The data presented in this study are available on request from the corresponding author.
